# Spatial-Temporal Clusters and Risk Factors of Hand, Foot, and Mouth Disease at the District Level in Guangdong Province, China

**DOI:** 10.1371/journal.pone.0056943

**Published:** 2013-02-21

**Authors:** Te Deng, Yong Huang, Shicheng Yu, Jing Gu, Cunrui Huang, Gexin Xiao, Yuantao Hao

**Affiliations:** 1 Department of Medical Statistics and Epidemiology and Health Information Research Center, School of Public Health, Sun Yat-sen University, Guangzhou, China; 2 Center for Public Health Surveillance and Information Service, Chinese Center for Disease Control and Prevention, Beijing, China; 3 Center for Environment and Population Health, School of Environment, Griffith University, Brisbane, Queensland, Australia; National Institutes of Health, United States of America

## Abstract

**Objective:**

Hand, foot, and mouth disease (HFMD) has posed a great threat to the health of children and become a public health priority in China. This study aims to investigate the epidemiological characteristics, spatial-temporal patterns, and risk factors of HFMD in Guangdong Province, China, and to provide scientific information for public health responses and interventions.

**Methods:**

HFMD surveillance data from May 2008 to December 2011were provided by the Chinese Center for Disease Control and Prevention. We firstly conducted a descriptive analysis to evaluate the epidemic characteristics of HFMD. Then, Kulldorff scan statistic based on a discrete Poisson model was used to detect spatial-temporal clusters. Finally, a spatial paneled model was applied to identify the risk factors.

**Results:**

A total of 641,318 HFMD cases were reported in Guangdong Province during the study period (total population incidence: 17.51 per 10,000). Male incidence was higher than female incidence for all age groups, and approximately 90% of the cases were children 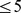 years old. Spatial-temporal cluster analysis detected four most likely clusters and several secondary clusters (*P*<0.001) with the maximum cluster size 50% and 20% respectively during 2008–2011. Monthly average temperature, relative humidity, the proportion of population 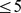 years, male-to-female ratio, and total sunshine were demonstrated to be the risk factors for HFMD.

**Conclusion:**

Children 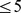 years old, especially boys, were more susceptible to HFMD and we should take care of their vulnerability. Provincial capital city Guangzhou and the Pearl River Delta regions had always been the spatial-temporal clusters and future public health planning and resource allocation should be focused on these areas. Furthermore, our findings showed a strong association between HFMD and meteorological factors, which may assist in predicting HFMD incidence.

## Introduction

Hand, foot, and mouth disease (HFMD), mainly caused by the enteroviruses virus (especially coxsackievirus A16 and enterovirus 71), has resulted in major outbreaks across the world in the past three decades [1]. The clinical presentations of HFMD are characterized by fever and vesicular exanthema mostly in hands, feet and oral mucosa [2]. The disease is usually mild and self-limiting, but sometimes serious neurological and cardiopulmonary complications may occur in HFMD outbreaks, particularly when the causative virus is enterovirus 71 [3], [4].

Recent epidemics have tended to be located in the Asian Pacific regions. In 2008, a large wave of HFMD epidemics occurred in mainland China, Taiwan, Malaysia, Singapore, Hong Kong, etc. In mainland China, epidemics started in Fuyang City, Anhui Province, resulting in 353 severe cases and 22 deaths, and then rapidly developed into a national-scale epidemic, covering 28 provinces within 3 months with 345,159 reported cases (accounting for 70.59% of the total reported cases of the year) [5], [6]. The Chinese Ministry of Health (MOH) listed HFMD as a notifiable Class-C communicable disease since May 2008 [7], [8]. According to the national network’s surveillance data, a total of 5,031,044 cases were officially reported in China during May 2008 to December 2011.

Guangdong, the largest southern province in China with a subtropical climate, accounted for 12.75% of all reported HFMD cases. In 2008, the number of reported HFMD cases in Guangdong Province was 47,660. This number almost doubled in 2009 (92,998 reported cases), was five-fold in 2010 (230,978 reported cases), and was six-fold in 2011 (269,682 reported cases). These numbers were more than four-fold of the national average level: in 2010 the average reported cases for each province were 57,246 and in 2011 the number was 52,381. The reasons for the high incidence in Guangdong Province might be due to its subtropical climate with high temperature and high humidity [9] and other demographic features such as greater population density, sex ratio, etc [10], [11].

Until now, effective chemoprophylaxis or vaccination approaches for dealing with HFMD are still not available. Epidemiological surveillance and an improved understanding of the spatial clustering of HFMD may provide useful insights into local epidemic control and resource allocation. Taking corresponding measures for high-risk populations during HFMD outbreaks can effectively reduce the incidence of HFMD. Therefore, we conducted this study to analyze the epidemic characteristic of HFMD in Guangdong Province, detect spatial-temporal clusters, and explore risk factors of HFMD for further public health interventions.

## Materials and Methods

### Study Area

Guangdong Province, situated in north latitude 20.15 N to 25.51 N and east longitude 109.75E to 117.33E, has a population of 10.3 million (from 2010 census data). It performs complex landforms through the latitude direction: a series of mountains are located in the province from northeast to southwest, and the eastern Pearl River Delta region is adjacent to the South China Sea Coast. In general, it is a low-latitude, high temperature and high humidity area.

**Figure 1:**
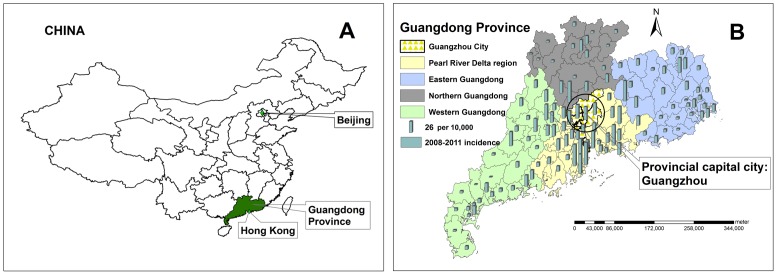
Geographic location of A) Guangdong Province in China and B) the four parts of Guangdong Province including the capital city: Guangzhou.

According to the characteristics of the natural landscape and economic development, Guangdong Province can be divided into four parts: the Pearl River Delta region (including the capital city Guangzhou), northern Guangdong mountainous and hilly, western Guangdong mesa, and eastern Guangdong mountainous and coast. The Pearl River Delta region (including the capital city Guangzhou) has a higher level of economic development as compared to the rests, accounting for 80% of GDP in Guangdong Province with less than 50% population (Figure 1).

### Surveillance Data of Hand, Foot, and Mouth Disease

Data of daily reported HFMD cases in Guangdong Province from May 1, 2008 to December 31, 2011 were obtained from the National Center for Public Health Surveillance and Information Services, China Center for Disease Control and Prevention (China CDC). The date was date of symptom onset, and every district were required to report HFMD cases daily via the web-based surveillance system with unified format, including the information of name, sex, age, address, date of symptom onset, etc. The clinical criteria for diagnosis of HFMD cases was provided in a guidebook published by the MOH in 2008 [7], in which patients were defined as HFMD with occurrence of the following symptoms: fever, papules and herpetic lesions on the hands or feet, rashes on the buttocks or knees, inflammatory flushing around the rashes and little fluid in the blisters, sparse herpetic lesions on oral mucosa.

### Meteorological Data

Monthly average temperature, monthly average maximum temperature, monthly average minimum temperature, monthly average relative humidity, monthly cumulative rainfall, monthly total sunshine and monthly average wind speed data for each district/county were obtained from the China Meteorological Data Sharing Service System (http://cdc.cma.gov.cn/). Complete meteorological data was available from May 2008 to May 2010 (Figure S1).

### Descriptive Epidemiology Analysis

Descriptive analysis was conducted by year to describe the demographic characteristics of reported HFMD cases. Graphs on the monthly number of reported HFMD cases and monthly distribution of the enteroviruses were drawn to show the seasonality of HFMD, cyclical patterns and the predominant circulating enteroviruses. All children were divided into four groups: live at home, in kindergarten, in primary school and others.

### Spatial-temporal Clusters

SaTScan™ software, version 9.1 (http://www.satscan.org/), using the Kulldorff method of retrospective space-time scan statistic based on a discrete Poisson model was used to detect HFMD clusters in individual districts/counties during the study period [12].

The space-time scan statistic is defined by a cylindrical window with a circular geographic base and with height corresponding to time [13]. The base and the height of the windows are in dynamic changes in order to detect possible spatial-temporal clusters. The base is centered around one of several possible centroids located throughout the study region, with the radius of the base varying continuously according to the population range of the area, from zero to the maximum cluster size of the total population who might be at risk. As there is no consensus on the maximum cluster size setting [14], [15], we performed cluster analysis with the default maximum spatial cluster size 50% of the population and again with a smaller maximum cluster size of 20% to look for possible sub-clusters [14], [15], [16]. The height reflects any possible time interval of less than or equal to half the total study period (default setting). The window is then moved in space and time, so that all possible geographic location and size could be checked.

The difference of the incidence inside and outside the windows was calculated by Statistic Log Likelihood Ratio (*LLR*):
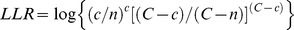



Where *C* denotes the total number of cases; *c* is the number of actual cases in the window; *n* is the number of expected cases in the window. The scan window with the largest *LLR* value is defined as most likely cluster; other scan windows that the *LLR* values are statistically significant are defined as secondary clusters. The relative risk (*RR*) of the incidence inside and outside the window is considered statistically significant if the *P* value is less than 0.05, which is evaluated by a Monte Carlo simulation [12].

In this study, spatial units referred to the 123 districts/counties located in Guangdong Province. Scan timeframe was set to be one year to observe the cluster changes and control the time trends in the whole study period [17].

### Spatial Paneled Model

We examined the role of different meteorological and demographic factors using a spatial panel model [18], [19], [20], [21]:
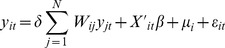
Where *i* and *t* are index for cross-sectional dimension (spatial units) and time dimension (time periods), respectively; 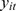 is the log transformation of the number of cases at *i* and *t*; *X* is the group of explanatory variables; 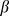 is the vector of regression coefficients that explain the relationship between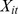 and 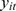; 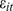 is an independently and identically distributed error term with zero mean and variance 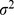, and 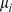 captures the spatial-specific effects whose omission could bias the estimates. 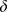 is the spatial autoregressive coefficient, reflecting the neighborhood effects between each spatial unit, and the normal range is 0 to 1 (the higher the value is, the stronger the neighborhood effects are).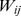 is a spatial weights matrix (SWM). As for regional data, if the region *i* is adjacent to the region *j*, 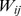 would be 1; otherwise, it would be 0.

In this study, we used the population size as the offset and nine independent variables from May 2008 to May 2010 were selected, including seven meteorology factors: monthly average temperature, monthly average maximum temperature, monthly average minimum temperature, monthly average relative humidity, monthly cumulative rainfall, monthly total sunshine, monthly average wind speed and two socio-demographic factors: proportion of population 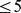 years old [22]and male-to-female ratio for each district/county. In consideration of the incubation period of enteroviruses and the potential delay in being aware of sickness, we examined the effect of meteorological variables with moving average of 0- and 1-month lags. For example, rainfall with moving average of 0- and 1-month lags refers to 2-month average of rainfall in the current and previous month. Indicators variables for years were included in the model to allow for long-term trends and inter-annual variations. Sine [Sin(2πt/12)] and cosine [Cos(2πt/12)] functions with a period of 12 months were used to control seasonal variation. A backward stepwise method was used in the regression model, and we reported the coefficient and the 95% confidence interval of each significant parameter. The model estimation was performed in MATLAB R2010a.

## Results

### Demographic Characteristics

Between May 1, 2008 and December 31, 2011, there are a total of 641,318 HFMD cases reported to the China National Public Health Surveillance System of Guangdong Province. Of these cases, 2,812 (0.44%) were severe cases. The incidence rates markedly increased (cox-stuart trend test, *P*<0.05) by year with 7.5 per 10,000 in 2008, 9.8 per 10,000 in 2009, 23.5 per 10,000 in 2010 and 26.3 per 10,000 in 2011.

The number of children 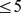 years old accounted for the largest proportion (from 87.5% to 93.3%) of all reported HFMD cases in the four-year study period. The incidence rate was highest in the 1- year age group, and this rises was the steepest from 178.1 per 10,000 in 2008 to 823.4 per 10,000 in 2011. There was a clear upward trend in the proportion of HFMD cases in 0-, 1- and 2- age groups. However, a downward trend in the proportion of cases in 3-, 4-, 5- and 10- age groups was observed during the period of study, meaning that younger age children were more vulnerable to HFMD (Table 1).

**Table 1:** Demographic characteristics of reported HFMD cases in Guangdong Province, China, 2008–2011

	2008		2009		2010		2011	
Case number	47,660		92,998		230,978		269,682	
**Age (years old)**	Incidence per 10,000	Proportion	Incidence per 10,000	Proportion	Incidence per 10,000	Proportion	Incidence per 10,000	Proportion
0-	82.1	12.4	123.8	15.0	293.2	15.3	373.3	14.7
1-	178.1	26.1	238.0	27.9	573.3	29.0	823.4	30.8
2-	159.8	22.4	223.5	25.0	440.0	21.2	640.1	22.7
3-	133.1	17.4	163.7	17.0	369.9	16.5	481.1	15.9
4-	72.2	9.1	73.6	7.3	196.0	8.3	251.2	7.8
5-	12.9	10.3	10.8	6.4	32.1	7.9	39.7	6.6
10-	0.2	2.2	0.1	1.3	0.5	1.8	0.4	1.5
**Gender**								
Male	9.8	65.9	12.5	65.5	29.7	64.6	32.6	64.7
Female	5.3	34.1	6.9	34.5	17.1	35.4	19.4	35.3
Sex ratio	1.84∶1		1.81∶1		1.74∶1		1.68∶1	
**Children groups**								
Live at home	–	67.3	–	72.2	–	74.0	–	79.2
In kindergarten	–	27.2	–	24.3	–	21.9	–	17.6
In primary school	–	4.8	–	2.8	–	3.5	–	2.6
Others	–	0.7	–	0.7	–	0.6	–	0.6
**Total**	7.5	100.0	9.8	100.0	23.5	100.0	26.3	100.0

The majority (approximately 65%) of HFMD cases were boys and the male-to-female incidence ratio were 1.84∶1 in 2008, 1.81∶1 in 2009, 1.74∶1 in 2010, and 1.68∶1 in 2011 respectively, *P*<0.001. The incidence in male was higher than that in female. In the four-year study period, children live at home were the predominant group of HFMD cases. The proportion of cases in this group increased from 67.3% in 2008 to 79.2% in 2011. Nevertheless, a gradual decline in the proportion of HFMD cases in institutional children and student groups was observed.

### Seasonal Pattern

**Figure 2:**
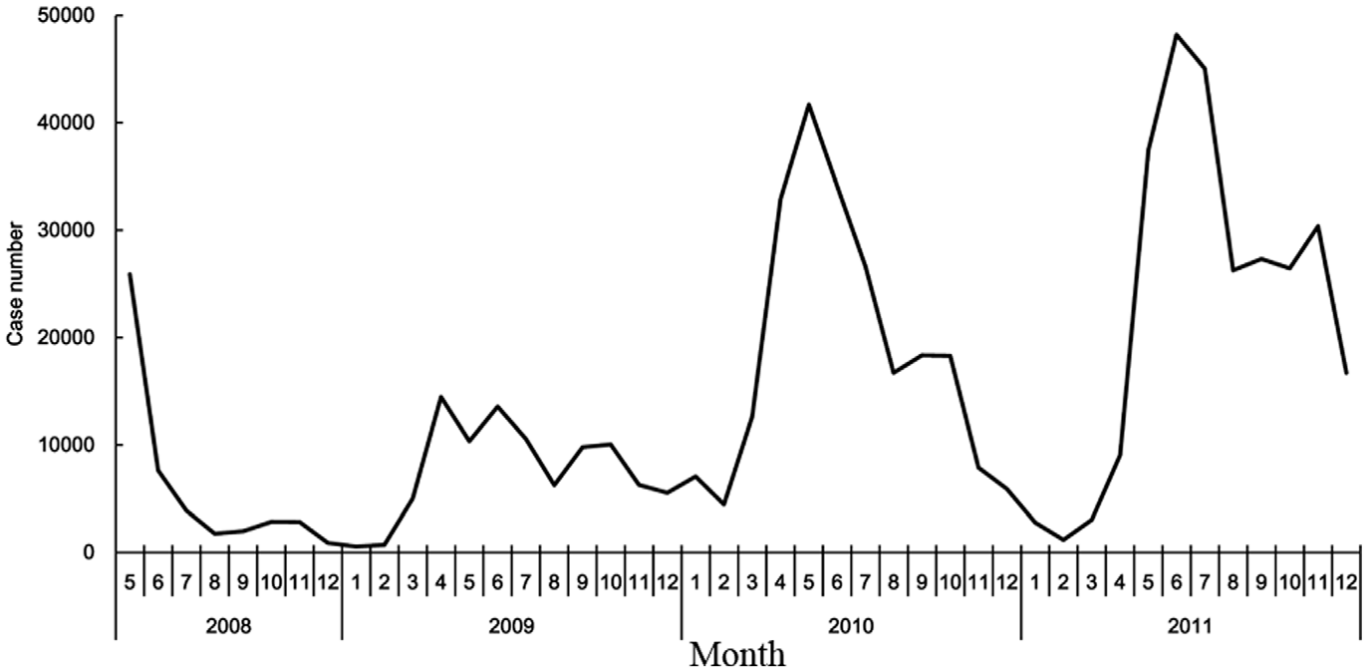
Monthly reported cases of HFMD in Guangdong Province, China, 2008–2011.

During the four-year study period, a summer peak was observed in May and June with a second smaller peak in October and November except year 2009, when the peak appeared in April happened to coincide with the influenza (H1N1) pandemic period. Two epidemic troughs occurred during the summer and winter school holidays around the same time in annual February and August (Figure 2).

### Laboratory Detection

**Figure 3:**
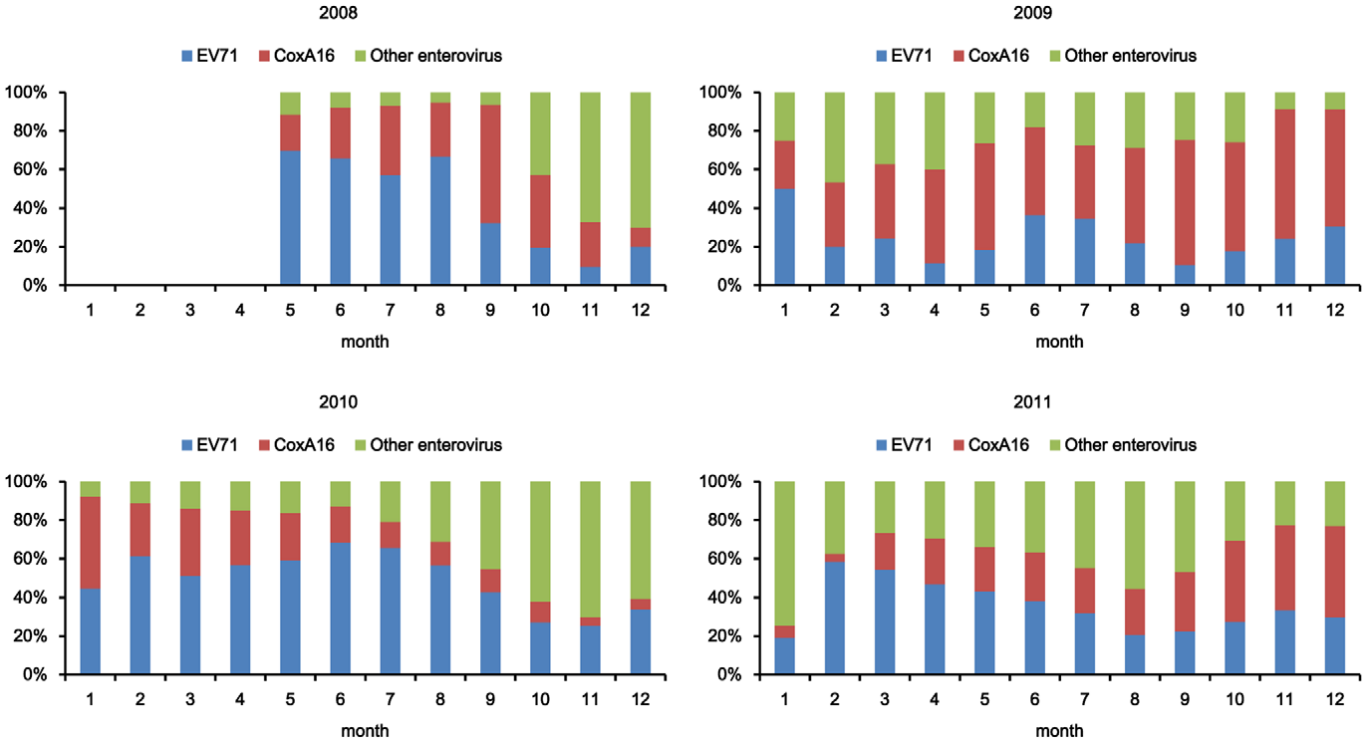
Monthly distribution of EV71, CoxA16 and other enterovirus in Guangdong Province, China, 2008–2011.

The majority of the enteroviruses isolated from the HFMD cases were coxsackievirus A16 (CoxA16) in 2009, while enterovirus 71 (EV71) was predominant in 2008 and 2010. However, CoxA16 and EV71 were almost equally distributed in 2011 (Figure 3).

### Spatial-temporal Clusters

**Figure 4:**
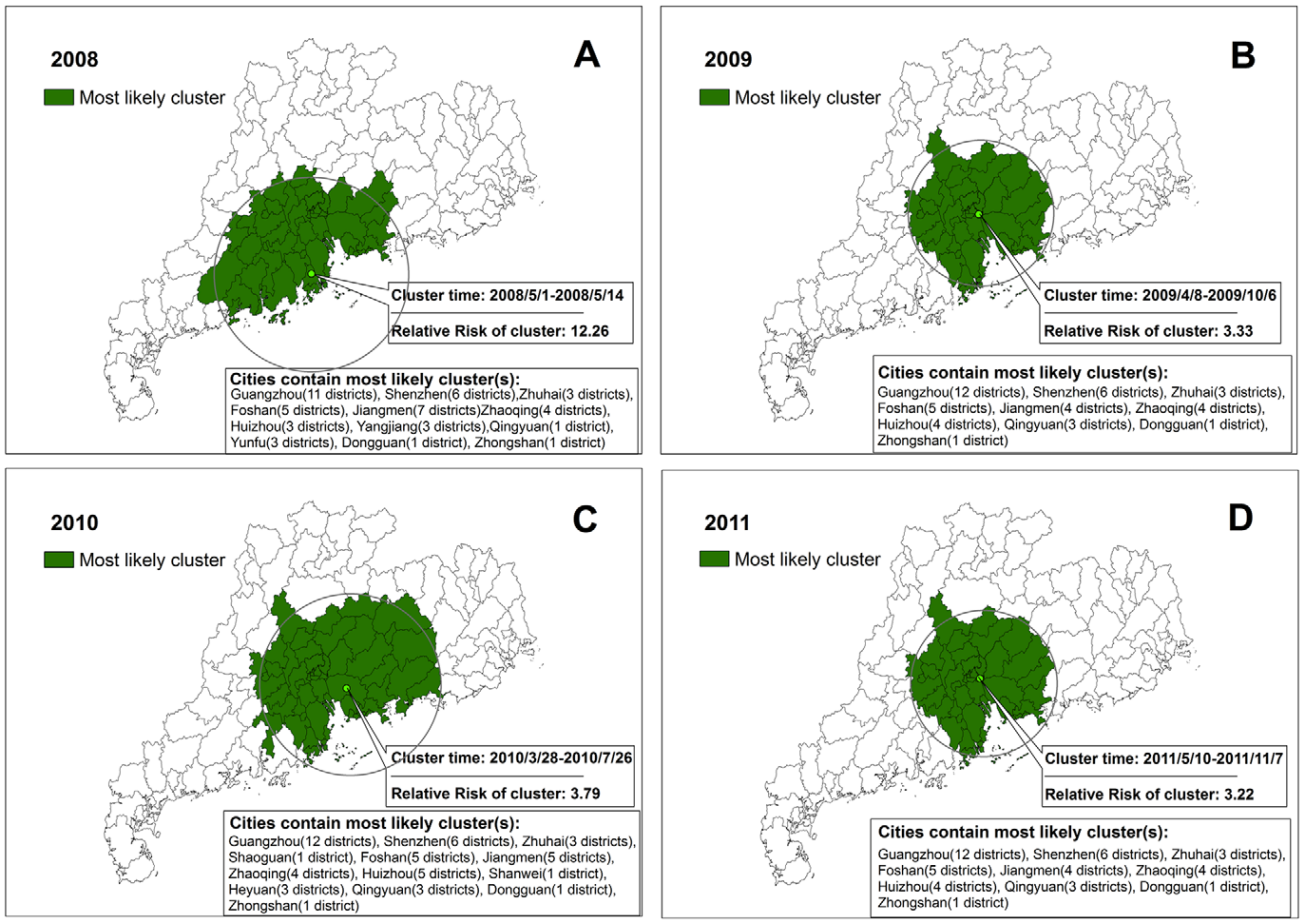
Spatial-temporal clusters of HFMD in Guangdong Province, China, in A) 2008, B) 2009, C) 2010 and D) 2011, setting 50% as the maximum cluster size. Dark green represents the most likely clusters. Annotations with the cluster time, *RR* value and cities contain the most likely clusters are presented by year.

Using the maximum spatial cluster size of 50% of the total population, spatial cluster analysis identified a most likely cluster that included 48 geographic districts/counties in 2008, of which the cluster center was (22.23N, 113.25E) and the cluster radius was 163.17 km. The cluster time was May 1 in 2008 to May 14 in 2008, and the average annual incidence rate inside the window was 84.14 per 10,000 with the relative risk value (*RR*) 15.96 (*P*<0.001). Other three most likely clusters could be similarly found from 2009 to 2011with almost the same cluster center and cluster radius (Table 2 and Figure 4).

**Table 2:** The most likely clusters of HFMD in Guangdong Province, China, 2008–2011 (setting 50% as the maximum cluster size).

Scan timeframe	Cluster time	Cluster center/Radius	Annual cases/10000	*LLR*	*RR*	*P*
2008/5/1–2008/12/31	2008/5/1–2008/5/14	(22.23N, 113.25E)/163.17 km	84.14	25169.84	15.96	<0.001
2009/1/1–2009/12/31	2009/4/8–2009/10/6	(23.10N, 113.48E)/119.57 km	21.16	15869.52	3.33	<0.001
2010/1/1–/12/31	2010/3/28–2010/7/26	(22.94N, 113.88E)/151.31 km	60.64	42847.38	3.79	<0.001
2011/1/1–2011/12/31	2011/5/10–2011/11/7	(23.10N, 113.48E)/119.57 km	54.20	44180.26	3.22	<0.001

Note: ‘Scan timeframe’ means the boundary of time points put into the scanning analysis, and the ‘Cluster time’ means the boundary of time points identified by the scanning analysis.

**Figure 5:**
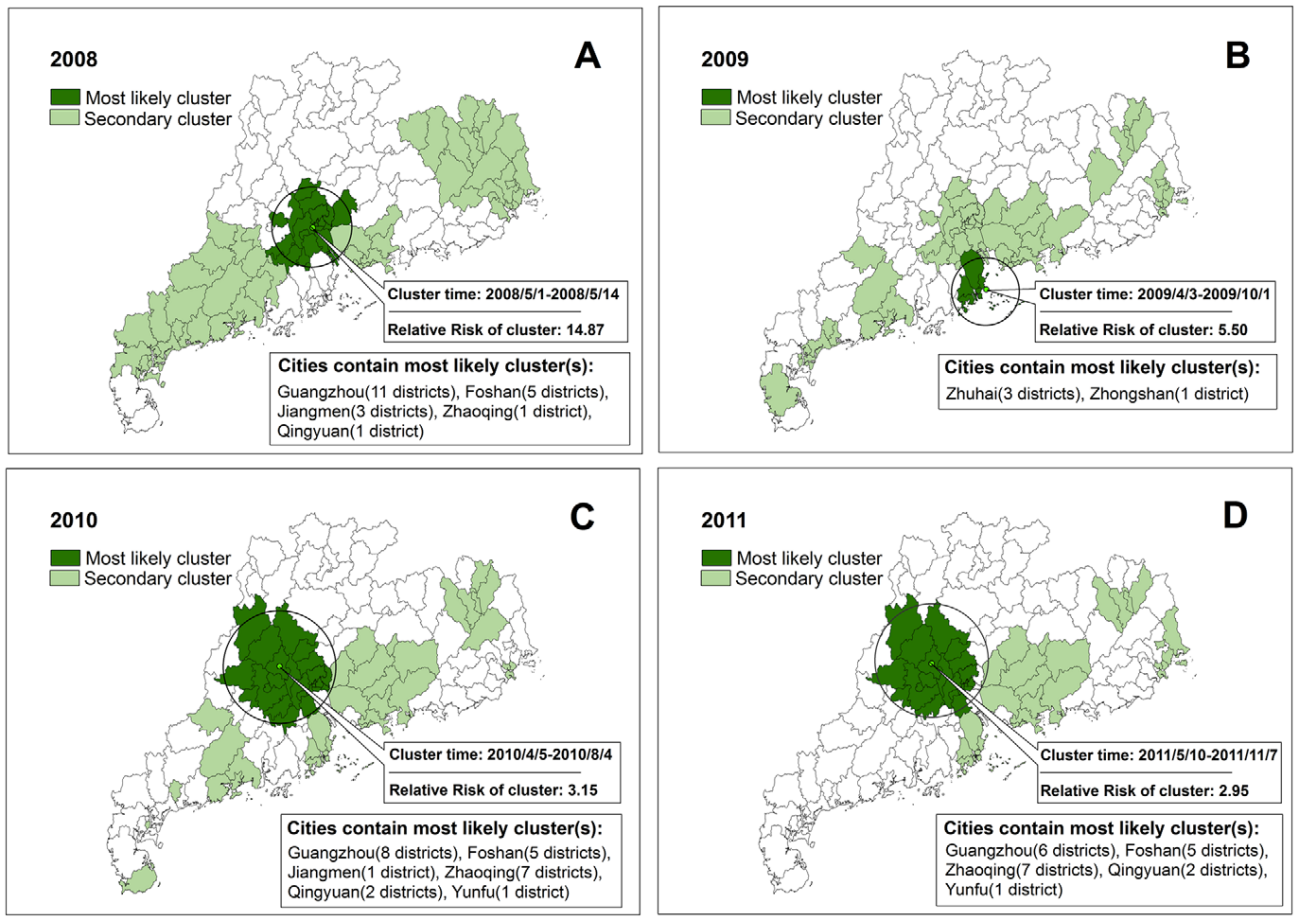
Spatial-temporal clusters of HFMD in Guangdong Province, China, in A) 2008, B) 2009, C) 2010 and D) 2011, setting 20% as the maximum cluster size. Dark green represents the most likely clusters and light green represents the secondary clusters. Annotations with the cluster time, *RR* value and cities contain the most likely clusters are presented by year.

To investigate the possibility of smaller clusters, the same analysis was performed with a modification of the maximum spatial cluster size 20% of the total population. One most likely cluster and three secondary clusters could be found in 2008. The most likely cluster included 21 geographic districts/counties, with the cluster center (23.09N, 113.22E) and cluster radius 64.16 km. The cluster time was May 1 in 2008 to May 14 in 2008, and the average annual incidence rate inside the window was 96.98 per 10,000 with the relative risk value (*RR*) 14.87 (*P*<0.001). Similarly, we could find one most likely cluster and several secondary clusters in 2009, 2010, and 2011respectively. The cluster centers were almost the same within the capital city Guangzhou and its neighboring areas except 2009: the most likely cluster areas in 2009 were Zhuhai and Zhongshan (Table 3 and Figure 5).

**Table 3:** The most likely clusters of HFMD in Guangdong Province, China, 2008–2011 (setting 20% as the maximum cluster size).

Scan timeframe	Cluster time	Cluster center/Radius	Annualcases/10000	*LLR*	*RR*	*P*
2008/5/1–2008/12/31	2008/5/1–2008/5/14	(23.09N, 113.22E)/64.16 km	96.98	11542.28	14.87	<0.001
2009/1/1–2009/12/31	2009/4/3–2009/10/1	(22.21N, 113.62E)/42.37 km	48.99	8302.80	5.50	<0.001
2010/1/1–2010/12/31	2010/4/5–2010/8/4	(23.43N, 112.68E)/89.53 km	64.24	17179.61	3.15	<0.001
2011/1/1–2011/12/31	2011/5/10–2011/11/7	(23.43N, 112.68E)/82.25 km	64.64	23793.36	2.95	<0.001

Note: ‘Scan timeframe’ means the boundary of time points put into the scanning analysis, and the ‘Cluster time’ means the boundary of time points identified by the scanning analysis.

### Risk Factors

The results of spatial panel model based on the variables mentioned before are presented in Table 4. The coefficients of the spatial dependence are highly significant in the models with δ of 0.2780 (*P*<0.001), indicating the presence of neighborhood effects. Monthly average temperature, monthly average relative humidity, proportion of population 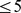 years and male-to-female ratio are positively associated with the HFMD incidence rate, while monthly total sunshine is negatively associated with the HFMD cases at the county level after controlling for the spatial effect. In addition, cumulative rainfall and wind speed were not statistically significant. The *R* ^2^ value was 0.67, indicating that the meteorology factors and socio-demographic factors could explain 67% variation of the HFMD incidence, including the spatial neighborhood effects.

**Table 4:** Spatial panel model using meteorological and demographic factors with moving average of 0- and 1-month lags on HFMD incidence in Guangdong Province, China, 2008–2011.

Variables	Coefficient	*S.E*	95% *CI*		*t*	*P*
			Lower	Upper		
Temperature (°C)	0.0918	0.0247	0.0434	0.1403	3.72	<0.001
Relative humidity (%)	0.0383	0.0080	0.0227	0.0539	4.80	<0.001
Total sunshine (h)	−0.1666	0.0386	−0.2422	−0.0910	−4.32	<0.001
Proportion of population 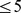 years (%)	0.6420	0.1086	0.4292	0.8549	5.91	<0.001
Male-to-female ratio (×100)	0.3908	0.1731	0.0516	0.7300	2.26	0.023
Spatial weight	0.2780	0.0216	0.2357	0.3203	12.89	<0.001

*R* ^2^ = 0.67.

## Discussion

In our study, we observed that children 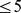 years old accounted for most of the HFMD cases during the study period in Guangdong Province, with a peak incidence at 1 year of age group, which was similar to other studies [23], [24], [25], [26], [27], [28], [29], [30]. This can be related to the differences in serum antibodies in different age groups. A recent seroepidemiological study showed that the level of maternal antibody titers declined markedly during the first 7 month and increased significantly from month 12 to months 27–38, although it stayed at a relatively low level [31]. Another study reported that over 50% of children aged 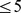 years had no neutralizing antibody against EV71 and CoxA16 [32].

The proportion of cases in those aged 0–2 years increased over time, while there was a gradual decline in the proportion of HFMD cases in those aged more than 3 years. This is consistent with our findings of an upward trend in proportion of children live at home, and of a downward trend in proportion of children in kindergartens and primary schools. A possible reason was that the Chinese MOH and local governments have initiated several measures to control the epidemic of HFMD in institutional settings since 2008, such as disinfection of toys, sanitary products and tableware, morning check, hand-washing intervention, case isolation system and school closure [33]. Consequently, the fact that the proportion of institutional HFMD cases decreased markedly could be due to the implementation of these strategies for reducing the spread of HFMD in children in kindergarten and school children, although the incidence of HFMD in all age groups increased annually over the past four years. We found that boys were more susceptible to enterovirus than girls, which is consistent with the previous studies [34]. Boys may have more physical activities than girls, which could lead to more contact favoring the spread of HFMD.

The seasonality of HFMD detected in Guangdong showed that there was a larger seasonal peak occurred in the late spring/early summer, along with a smaller peak in late autumn/early winter. This was consistent with the findings in other Asian areas such as Singapore, Malaysia, Hong Kong and Taiwan [27], [28], [35]. In addition, two epidemic troughs in Guangdong Province were found annually in the same time in summer and winter holidays when there was less contact in the children.

Two major isolated enteroviruses of HFMD cases in Guangdong Province were EV71 and CoxA16, and the predominant virus varied each year. EV71 was the predominant virus circulating during the epidemic in 2008 and re-emerged in 2010 and 2011 with co-circulation of CoxA16, while CoxA16 was the predominant virus detected in 2009. It was presumed that the periodicity could be due to the accumulation of susceptible children between epidemics, the virus variation and the effective interventions. However, it is hard to ascertain the cycle of the virus during the 4-year period and it requires a longer monitoring of HFMD to determine the cyclical pattern of EV71 and CoxA16 in Guangdong Province.

During the four-year study period, HFMD incidence in Guangdong Province increased from 7.5 per 10,000 in 2008 to 26.3 per 10,000 in 2011. This could be due to the real high incidence of HFMD in 2010 and 2011, as well as the improved diagnostic capacity and enhanced supervision of HFMD. Besides, incomplete data in 2008 and pandemic of H1N1 in 2009 might influence the trends. During the H1N1 influenza epidemic in 2009, the transmission of respiratory viruses among children in Guangdong Province was greatly reduced by massive use of face masks, school closures and reduction of outdoor activities. These measures could also prevent the transmissions of HFMD.

Kulldorff scan statistic is widely used in the detection of spatial-temporal clusters of infectious diseases, cancer, birth defects and other diseases [36]. There is no pre-selection bias because the clusters are searched with no prior hypothesis on their location, size or time period, so it can effectively utilize the time and spatial information. Besides, Monte-Carlo randomization method for hypothesis testing gives the empirical joint distribution of the statistics and hence accounts for the correlation among the statistics, delivering a *P* value after taking into account multiple testing [37]. However, selection of the least spatial scale might influence the scanning results, which was called the ecological fallacy [38]. In our study, we control the research spatial scale to a small level (district/county level) to reduce the ecological fallacy. However, we could not get more precise data at a smaller level (for example, village/town level data). Despite this, it was still important to point out that larger units might bias the results.

Another issue was that the definition of maximum cluster size would affect the scanning results. In our study, we chose both 50% and 20% of the total population at risk as the maximum cluster size to detect spatial-temporal clusters and possible sub-clusters (Table 2 and Table 3, Figure 4 and Figure 5). Setting 50% of the population at risk as the maximum cluster size, we could get almost the same cluster centers with large cluster radius covering approximately one third of the districts/counties in Guangdong Province. Setting 20% of the population at risk as the maximum cluster size, we could find one most likely cluster and several sub-clusters for each year. In 2008, 2010 and 2011, the cluster centers were almost the same located in the provincial capital city Guangzhou and its neighboring areas. Some cities like Foshan, Zhaoqing, Qingyuan, Jiangmen, and Yunfu were detected to be the most likely clusters for each year. However, the scanning result in 2009 was different: two cities (Zhuhai and Zhongshan) with high average annual incidence rate (48.99 per 10,000) and *RR* value (5.50, *P*<0.001) were detected to be the most likely clusters. These two cities were adjacent to the South China Sea Coast instead of the provincial capital city Guangzhou. The results reminding us that HFMD prevention and control measures should be focused on Guangzhou and its neighboring areas as well as the two cities Zhuhai and Zhongshan to make cost-effectiveness maximized.

When setting 20% as the maximum cluster size, we could find amounts of secondary clusters in Guangdong Province in the four years, covering almost one half of the whole study area with different cluster centers and cluster time. *RR* ranged from 1.54 to 14.11(*P*<0.001), indicating that many areas in Guangdong Province had suffered a different degree of outbreaks from 2008 to 2011 (Figure 5). Targeting prevention strategies at areas of highest risk can potentially increase the interventions’ effectiveness.

Spatial dependence might have existed between the observations at each unit at each time when data were geo-referenced, especially in infectious disease monitoring data [39]. Spatial panel model is typically used to analyze data containing time-series observations of a number of spatial units (counties, regions, states, counties, etc.). As noted by Elhorst [19], [20], spatial panel models were more informative and contained more variation and less co-linearity among variables than purely cross-sectional models or time-series models. Taking into account the meteorological factors and demographic factors, the high incidence rate of HFMD cases for different districts/counties in Guangdong Province were associated with a higher average temperature, relative humidity, proportion of children 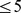 years, male-to-female ratio and lower total sunshine. Climate factors and demographic changes in Guangdong Province such as population structure, population growth and urbanization may be contributors affecting the epidemic situation of HFMD.

Some limitations are deserved to mention: 1) Study period. We collected reported HFMD cases of Guangdong Province from May 2nd 2008 to December 31st 2011 from China CDC surveillance network. However, the study period is not long enough. Further long-term surveillance data analysis is needed to identify the cyclical patterns of EV71 and CoxA16. 2) Spatial scale. We used district/county as the least spatial analysis unit (scale), which may lead to a modifiable areal unit problem (MAUP) which is a sub-class of ecological fallacy. If we could use a finer areal unit scale such as village/town in Guangdong Province, we may have obtained different results in spatial-temporal cluster detection and risk factor analysis [40]. 3) Lack of virus types’ information: It would be a good idea that if we could conduct the analyses stratified by virus types and year, however, we cannot since not every reported case has done a laboratory testing for disease pathogen. It should be considered in future study.

In conclusion, HFMD is a widespread infectious disease in Guangdong Province, which has posed a great threat to the health of children. A better understanding of the epidemic characteristic and spatial-temporal clusters of HFMD can help predict the epidemic trends and provide appropriate public health measures to make cost-effectiveness maximized. For example, health departments should pay close attention to the recurring clusters at the cluster time, strengthening disinfection and management. This may help us to control the HFMD prevalence and reduce its harm to people. Our study mainly focused on the descriptive analysis of the HFMD epidemic in Guangdong Province from 2008 to 2011, and further analysis was conducted to detect the spatial-temporal clusters and risk factors of HFMD based on Kulldorff scan statistic and paneled models. The results provides preliminary but fundamental information that may be useful to health authorities in helping cope with the HFMD transmission and target vulnerable populations.

## Supporting Information

Figure S1 **Time series of meteorological data in Guangdong Province, China, 2008–2011.** A) average temperature, B) total sunshine, C) average maximum temperature, D) cumulative rainfall, E) average minimum temperature, F) average relative humidity, G) average wind speed.
(TIFF)
