# Supplementary figures and images for: Spatial-Temporal Clusters and Risk Factors of Hand, Foot, and Mouth Disease at the District Level in Guangdong Province, China
Source: PLoS One. 2013 Feb 21;8(2):e56943. doi: 10.1371/journal.pone.0056943 (PMC3578924; doi:10.1371/journal.pone.0056943)

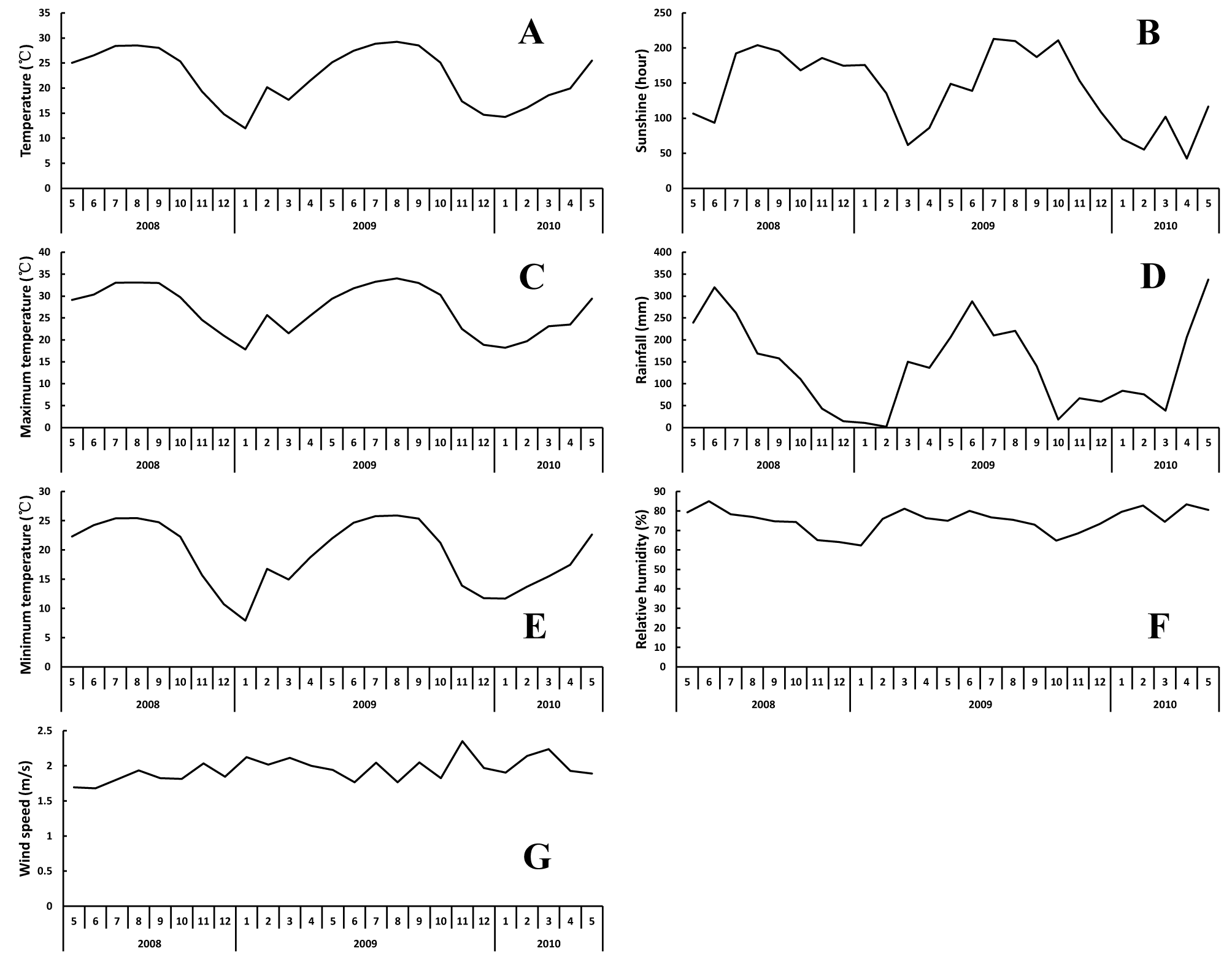

Supplement: Figure S1 — Time series of meteorological data in Guangdong Province, China, 2008–2011. A) average temperature, B) total sunshine, C) average maximum temperature, D) cumulative rainfall, E) average minimum temperature, F) average relative humidity, G) average wind speed. (TIFF) [file pone.0056943.s001.tiff]
